# Supplementary material for: Dynalign II: common secondary structure prediction for RNA homologs with domain insertions
Source: Nucleic Acids Res. 2014 Nov 21;42(22):13939–48. doi: 10.1093/nar/gku1172 (PMC4267632; doi:10.1093/nar/gku1172)
Supplement: SUPPLEMENTARY DATA [file supp_gku1172_nar-02021-z-2014-File012.zip › manual/RNA_class/html/graph_legend.html]

RNAstructure: Graph Legend

- Main Page
- Classes

# Graph Legend

This page explains how to interpret the graphs that are generated by doxygen.

Consider the following example:

```
/*! Invisible class because of truncation */
class Invisible { };

/*! Truncated class, inheritance relation is hidden */
class Truncated : public Invisible { };

/* Class not documented with doxygen comments */
class Undocumented { };

/*! Class that is inherited using public inheritance */
class PublicBase : public Truncated { };

/*! A template class */
template<class T> class Templ { };

/*! Class that is inherited using protected inheritance */
class ProtectedBase { };

/*! Class that is inherited using private inheritance */
class PrivateBase { };

/*! Class that is used by the Inherited class */
class Used { };

/*! Super class that inherits a number of other classes */
class Inherited : public PublicBase,
                  protected ProtectedBase,
                  private PrivateBase,
                  public Undocumented,
                  public Templ<int>
{
  private:
    Used *m_usedClass;
};
```

This will result in the following graph:

The boxes in the above graph have the following meaning:

- A filled gray box represents the struct or class for which the graph is generated.
- A box with a black border denotes a documented struct or class.
- A box with a grey border denotes an undocumented struct or class.
- A box with a red border denotes a documented struct or class forwhich not all inheritance/containment relations are shown. A graph is truncated if it does not fit within the specified boundaries.

The arrows have the following meaning:

- A dark blue arrow is used to visualize a public inheritance relation between two classes.
- A dark green arrow is used for protected inheritance.
- A dark red arrow is used for private inheritance.
- A purple dashed arrow is used if a class is contained or used by another class. The arrow is labeled with the variable(s) through which the pointed class or struct is accessible.
- A yellow dashed arrow denotes a relation between a template instance and the template class it was instantiated from. The arrow is labeled with the template parameters of the instance.

---

Generated on Sun Jul 19 11:06:55 2009 for RNAstructure by 
 1.5.7.1
